# Ocular Surface Microbiota in Naïve Keratoconus: A Multicenter Validation Study

**DOI:** 10.3390/jcm12196354

**Published:** 2023-10-04

**Authors:** Carlos Rocha-de-Lossada, Cosimo Mazzotta, Federico Gabrielli, Filomena Tiziana Papa, Carmen Gómez-Huertas, Celia García-López, Facundo Urbinati, Rahul Rachwani-Anil, María García-Lorente, José-María Sánchez-González, Miguel Rechichi, Giovanni Rubegni, Davide Borroni

**Affiliations:** 1 Eyemetagenomics Ltd., 71–75, Shelton Street, Covent Garden, London WC2H 9JQ, UK; carlosrochadelossada5@gmail.com; 2 Ophthalmology Department, QVision, Vithas Almería, 04120 Almeria, Spain; 3 Ophthalmology Department, Hospital Regional Universitario Málaga, 29010 Malaga, Spain; facundou10@gmail.com (F.U.); glorentemaria@gmail.com (M.G.-L.); 4 Siena Crosslinking Center, 53100 Siena, Italy; cgmazzotta@libero.it; 5 Departmental Ophthalmology Unit, USL Toscana Sud Est l, 53100 Siena, Italy; 6 Postgraduate Ophthalmology School, University of Siena, 53100 Siena, Italy; giovannirubegni@gmail.com; 7 Biolab SRL, Laboratorio di Genetica e Genomica Molecolare, Largo degli Aranci, 9, 63100 Ascoli Piceno, Italy; federico.gabrielli@laboratoriobiolab.it (F.G.); ngs@laboratoriobiolab.it (F.T.P.); 8 Department of Ophthalmology, Hospital Universitario Virgen de las Nieves, 18014 Granada, Spain; carmen_8871@hotmail.com (C.G.-H.); cegarcilop@gmail.com (C.G.-L.); 9 Department of Ophthalmology, Hospital de Antequera, 29200 Malaga, Spain; rahul.medum@gmail.com; 10 Department of Physics of Condensed Matter, Optics Area, University of Seville, 41012 Seville, Spain; jsanchez80@us.es; 11 Centro Polispecialistico Mediterraneo, 88050 Sellia Marina, Italy; miguel.rechichi@gmail.com

**Keywords:** keratoconus, metagenomics, validation-study, microbiota

## Abstract

In the field of Ophthalmology, the mNGS 16S rRNA sequencing method of studying the microbiota and ocular microbiome is gaining more and more weight in the scientific community. This study aims to characterize the ocular microbiota of patients diagnosed with keratoconus who have not undergone any prior surgical treatment using the mNGS 16S rRNA sequencing method. Samples of naïve keratoconus patients were collected with an eNAT with 1 mL of Liquid Amies Medium (Copan Brescia, Italy), and DNA was extracted and analyzed with 16S NGS. The microbiota analysis showed a relative abundance of microorganisms at the phylum level in each sample collected from 38 patients with KC and 167 healthy controls. A comparison between healthy control and keratoconus samples identified two genera unique to keratoconus, Pelomonas and Ralstonia. Our findings suggest that alterations in the microbiota may play a role in the complex scenario of KC development.

## 1. Introduction

Keratoconus (KC) is an inflammatory bilateral but asymmetric corneal disease [1]. Characterized by progressive corneal thinning resulting in central or paracentral corneal protrusion, KC produces a progressive decrease in visual acuity [2]. Although it usually appears around the second decade of life, it has been observed that onset in childhood seems to be associated with more severe forms of KC and with a trend to progress with an inverse correlation between age and severity [3,4]. Different treatments have been proposed, highlighting corneal cross-linking (CXL) to prevent progression, intracorneal segments (ICRS) and Bowman layer transplantation to reshape the cornea [5,6,7], and corneal transplantation in the form of penetrating keratoplasty or deep anterior lamellar keratoplasty (DALK) for advanced stages of the disease [8].

The human body is populated by an enormous variety of bacteria, archaea, fungi, and viruses, which form a commensal, symbiotic, and pathogenic community known collectively as the human microbiome [9,10]. The estimated number of microorganisms is on the order of trillions, at least ten times more than the number of human cells.

A fundamental step was represented by the Human Microbiome Project (HMP) launched in 2008 by the National Institutes of Health of the United States, with the objective of revealing and characterizing the microbial populations of five main areas of the body, including the intestine, mouth, nose, skin and urogenital tract [11,12]. The HMP has revealed that the microbiome plays a prominent role in the pathogenesis of autoimmune diseases [13]. However, this project did not include the study of the eye or ocular surface.

Metagenomics is the science that deals with the genomic analysis of populations of microorganisms based on the development of metagenomic next-generation sequencing (mNGS) [14]. Ideally, mNGS can detect all microorganisms present in a sample, producing huge amounts of sequencing data that must be decoded [15], potentially improving the performance of pathology diagnosis since it is an inherently unbiased and hypothesis-free approach [14,16]. Broadly, there are two main approaches for using mNGS, each with different indications, advantages, and limitations: targeted amplification sequencing and whole-genome “shotgun” metagenomics.

Targeted amplification sequencing, as the name implies, involves primer-mediated amplification of specific genomic targets (e.g., 16S rRNA for bacteria [17,18] and 18S rRNA for eukaryotes) [19,20,21]. By contrast, shotgun mNGS involves indiscriminate amplification of all nucleic acids contained in biological samples and, as such, is a hypothesis-free and culture-independent approach to clinical diagnosis of the microorganisms present in a sample [22,23]. However, a consequence of this non-targeted approach in the case of samples with low biomass is that the vast majority of the readings are derived from the host.

Over the past decade, mNGS has been used as a diagnostic modality test of last resort to detect pathogens in patients with a variety of severe systemic diseases [24,25,26,27,28,29] or in whom conventional microbiology has failed to identify a particular infectious agent causing a particular disease [30,31,32,33].

In the field of Ophthalmology, the study of the microbiota and the ocular microbiome is gaining more and more weight in the scientific community. The ocular surface microbiota is characterized by commensal bacteria that theoretically do not cause infection or inflammation [34,35]. In fact, they contribute to the homeostasis of the ocular surface, together with a mechanism of immunological tolerance by the ocular surface structures [34,35]. A study with the largest sample of the healthy population has recently been published by our group, in which it was shown that the ocular microbiota of the healthy population is a microbiome of low diversity. The vast majority of the 137 samples analyzed were highly enriched for Staphylococcus, while other genera such as Bacillus, Pseudomonas, and Corynebacterium predominate in only a few of them. This study found an average of 88 genera with an average Shannon index of 0.65. In addition, the concept of eye community state type (ECST) was defined as a stratification of the healthy ocular population. Nine ECSTs were found; that is, it was shown that the healthy ocular population has nine different profiles [33].

The tear proteome of patients with KC has recently been identified and profiled using the mNGS shotgun approach, where the authors compared a group with KC and a healthy control group. In this study, the authors described a total of 232 proteins, of which 133 were expressed in samples from both groups, 41 were observed only in samples from the control group, and 58 were identified only in the tears of patients with KC [36]. Likewise, another recently published study evaluated ten patients with KC who underwent CXL and ten healthy controls who underwent photorefractive keratectomy using mNGS 16S rRNA. The authors found that at the genus level, the relative abundance rates of twenty bacteria were significantly different between KC and healthy corneas (*p* < 0.05). Aquabacterium was the most abundant genus in patients with KC, while Shigella was the most abundant genus in healthy controls. Alpha diversity parameters were lower in patients with KC, although the difference did not reach statistical significance (*p* > 0.05) [37]. However, to the best of our knowledge, no study has evaluated the surface microbiota of naïve KC patients, that is, without any surgical procedure.

The main aim of this study is to characterize the ocular microbiota of patients diagnosed with KC who have not undergone any prior surgical treatment using the mNGS 16S rRNA sequencing method. Secondly, we compared it with the microbiota of the previously described healthy ocular population.

## 2. Materials and Methods

This study was approved by the institutional review board of the Virgen de las Nieves University Hospital (Granada, Spain) with the code (Metagenomic/queratocono 1844-N-22). Patients were recruited in the Opthalmology department of Virgen de las Nieves University Hospital, Siena Crosslinking Center, Siena, Italy, and Centro Oculistico Borroni (Gallarate, Italy) from January–October 2022

This descriptive cross-sectional study followed the tenets of the Declaration of Helsinki, and informed consent was obtained from human subjects involved in the study.

The inclusion criteria were limited to those patients diagnosed with KC confirmed by a licensed expert corneal ophthalmologist through a clinical examination and corneal tomography with Pentacam^®^ (Oculus Optikgerate GmbH, Wetzlar, Germany). For this purpose, KC-selected patients should be naïve subjects, not using antibiotics or anti-inflammatory drops, not wearing contact lenses for at least four days before the visit, and 18 years or older. In these patients, the eye with more stage of KC, according to TKC and ABCD classification from Pentacam^®^, were selected.

Among the exclusion criteria were patients with KC who had been treated by CXL, ICRS, or any type of corneal transplant or the presence of any ophthalmic or systemic disease different from KC.

The sample size was evaluated with the GRANMO^®^ calculator (Institut Municipal d’Investigació Mèdica, Barcelona, Spain. Version 7.12). The two-tailed test was used. Alpha and beta risk were set at 5% and 20%, respectively. The estimated standard deviation (SD) of the differences was set at 4.17 diopters (based on López-López et al. [36] 1 SD of the primary variable), the expected minimum (maximum) keratometry difference was set at 3.0 diopters, and finally, the rate of loss to follow-up was set at 0.00. This achieved a recommended sample size of at least 29 subjects.

### 2.1. Sampling Technique, DNA Extraction, PCR Amplification, Library Preparation, and Amplicon Sequencing

Samples were collected with an eNAT with 1 mL of Liquid Amies Medium (Copan, Brescia, Italy). The eNAT was applied on the inferior surface of the eye and moved two times, “limbus to fornix to limbus”. Neither fluorescein nor anesthetic drops were used to avoid influences on the eye microbiota. Microbial DNA from keratoconus and healthy control samples were standardly isolated using QIAamp DNA Microbiome Kit (Qiagen, Hilden, Germany) following the manufacturer’s instructions.

The Microbial DNA was checked and quantified using Genomic DNA ScreenTape (Agilent Technologies, Santa Clara, CA, USA) at Agilent TapeStation 4150. DNA libraries were set with Ion 16S Metagenomics Kit (Thermo Fisher, Waltham, MA, USA). An input DNA amount of 0.5 ng was used for library preparation. Amplification was completed using two primer sets to amplify the hypervariable regions of the 16S rDNA gene. Amplified products were purified using AMPure XP beads (Beckman Coulter Agencourt, Thermo Fisher, Waltham, MA, USA) and end-repaired for barcode ligation. Later, libraries were verified for quality and quantified using Agilent High Sensitivity D1000 ScreenTape (Agilent Technologies, USA). Equimolar libraries were pooled together at the final concentration of 5 pM. Template preparations were performed with Ion Chef according to the Ion 540 Kit-Chef protocol (Thermo Fisher, Waltham, MA, USA). The amplicon libraries were sequenced on a 540-chip using the Ion Torrent S5 system (Thermo Fisher, Waltham, MA, USA) according to the supplier’s instructions. After sequencing, low-quality and polyclonal sequences were filtered out by the Ion software, and the gained data were submitted to the dedicated software for analysis.

Nuclease-free water and all the reagents engaged in the experiment were processed as a negative control. The contamination with extraneous bacterial DNA in DNA extraction kit reagents and the wider ambulatory and laboratory environment was minimized by collecting control and experimental samples at the same time, under the same conditions, and handled together.

### 2.2. Data Analysis

Raw reads were analyzed with GAIA (v 2.02) (https://metagenomics.sequentiabiotech.com, accessed on 30 August 2022) to obtain operational taxonomic unit (OTU) tables at different taxonomic levels. Gaia is an online, easy-to-use proprietary platform for microbiome analyses with a high prediction of 0.978 at the genus level The present study focused on genera. To filter putative false positives, only those genera supported by at least two reads in at least two samples were considered for the downstream analyses. The richness and Shannon alpha diversity metrics, as well as Bray–Curtis beta diversity values, Chao1 and Mann–Whitney, were computed with the R package phyloseq. Principal coordinate analysis (PCoA) was performed using the function plot_ordination from the R package phyloseq.

## 3. Results

### 3.1. Comparative Analysis of Microbiome Composition Reveals Significant Differences between Control and Keratoconus Groups at the Phylum Level

**Figure 1:**
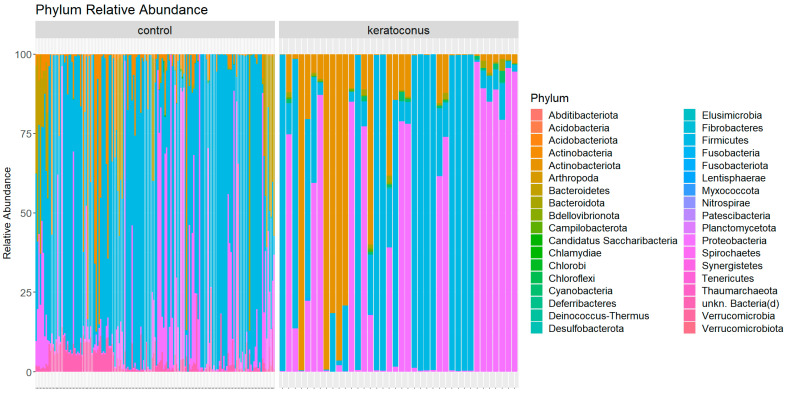
The relative abundance for the most common taxa at the phylum level and their percentage in each sample. The panel on the left shows healthy controls, and the panel on the right shows keratoconus samples. The most abundant phylum in the dataset is Firmicutes, which makes up 61.6% of the microbiota. This is followed by Proteobacteria (19.8%) and Actinobacteria (6.1%). However, the phylum unkn. Bacteria(d), with a relative abundance of 3.6%, is also notably abundant.

The microbiota analysis identified 36 phyla in all samples. Figure 1 shows the relative abundance of microorganisms at the phylum level in each sample collected from 38 patients with KC and 167 healthy controls.

Significant differences in the microbiota composition between the healthy control and the KC condition are detectable (Figure 1).

Our analysis examined the top ten most abundant phyla in the microbiota data from the control and KC groups. We found significant differences in the distributions of the relative abundance of several phyla between the two conditions. In particular, the phyla Actinobacteriota (0% in control vs. 18.68% in KC), Bacteroidota (0% in control vs. 0.51% in keratoconus), unkn. Bacteria(d) (4.43% in control vs. 0% in KC), Actinobacteria (7.60% in control vs. 0% in KC), and Bacteroidetes (5.97% in control vs. 0% in KC) showed significant differences between the two groups, as also indicated by the extremely low corrected *p*-values from the Mann–Whitney U tests (all *p*-values < 10^−15^). This suggests that these phyla’s relative abundance significantly varies between the control and KC conditions.

On the other hand, the phyla Firmicutes (65.84% in control vs. 42.99% in KC), Cyanobacteria (0% in control vs. 0.49% in KC), and Proteobacteria (15.97% in control vs. 37.08% in KC), despite being among the most abundant in both conditions, did not show a statistically significant difference between the control and keratoconus groups after adjusting for multiple comparisons. This suggests that the distributions of their relative abundance are not significantly different between the two conditions based on the tests performed.

### 3.2. Genus-Level Comparison Reveals Significant Differences in Microbiota Composition between Control and Keratoconus Groups

**Figure 2:**
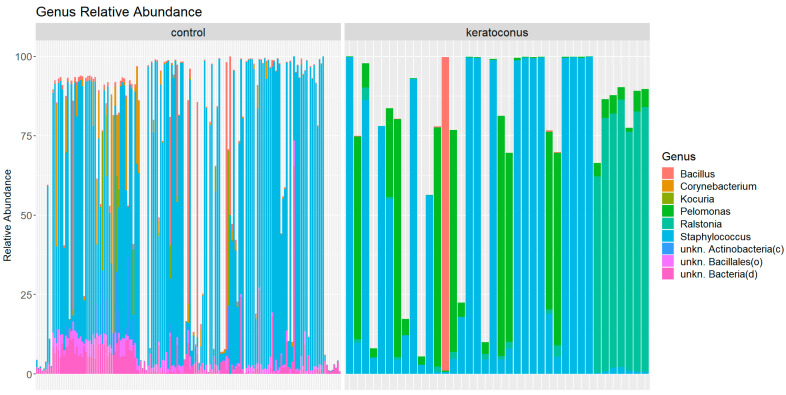
Microbiota composition of genera in healthy control and keratoconus samples. The panel on the left shows healthy controls, and the panel on the right shows keratoconus samples. The most represented genus was Staphylococcus in healthy control samples (79.05%), while in keratoconus samples, the most represented genera were Pelomonas (21.18%) and Ralstonia (19.11%).

We focused on the top 10 most abundant genera in the microbiota data from the healthy control and keratoconus groups (Figure 2). This approach enabled us to identify significant differences in the distributions of several genera between the two conditions. A comparison of genera between healthy control and keratoconus samples identified two genera unique to KC, Pelomonas (21.18%) and Ralstonia (19.11%), and five genera specific to healthy control: unkn. Bacteria(d) (6.56%), Corynebacterium (3.89%), unkn. Bacillales(o) (2.72%), Kocuria (1.71%), and unkn. Actinobacteria(c) (1.51%). The only genus, Bacillus, was represented in healthy controls and KC patients, 4.57% and 3.38%, respectively.

The extremely low corrected *p*-values from the Mann–Whitney U test (all *p*-values < 10 × 10^−10^) provide strong statistical evidence of these differences. The genus Pelomonas had a corrected *p*-value of approximately 2.56 ×10^−43^, indicating a significant difference in its distribution between the control and keratoconus groups.

Conversely, the genus Staphylococcus, despite being among the top 10 most abundant genera in both conditions (79.05% in control vs. 56.33% in KC), did not show a statistically significant difference in its distribution between the control and KC groups after adjusting for multiple comparisons (*p*-value approximately 0.929).

**Figure 3:**
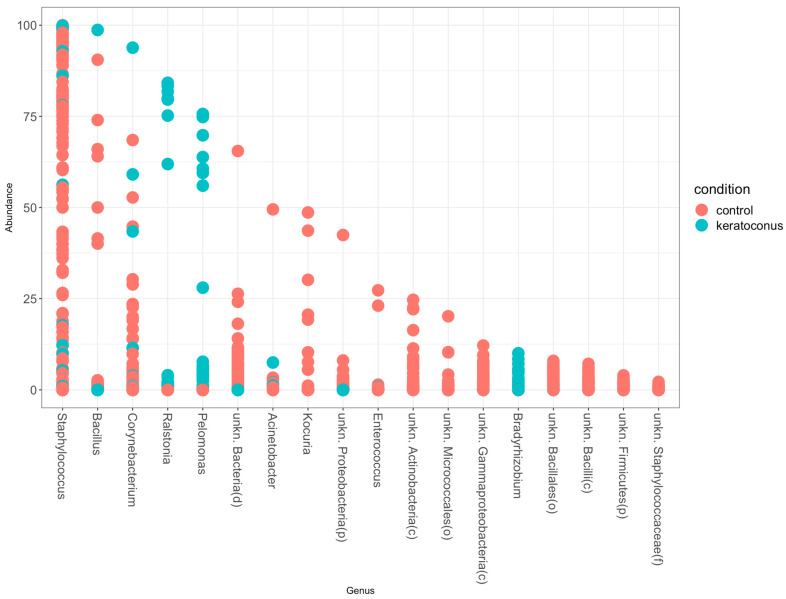
Staphylococcus abundance in the healthy control (red dots) and KC samples (green dots). Staphylococcus is the most abundant genus in the analyzed microbiota, representing 79.0% and 56.3% in the healthy control and KC groups, respectively.

Comparing the results with the healthy control and KC group (Figure 3), we observe that the genus Staphylococcus is the most abundant genus in both conditions. However, it makes up a larger proportion of the microbiota in the control group (79.05%) than in the KC group (56.33%). On the other hand, the genera Pelomonas and Ralstonia, which are among the most abundant in the KC group (making up 21.18% and 19.11% of the total relative abundance, respectively), are not among the top genera in the control group. This suggests a potential difference in the microbial communities between the two conditions. The genus Bacillus, which makes up 3.38% of the total relative abundance in the KC group, is also present in the control group, accounting for 4.57% of the total relative abundance. This indicates that the relative abundance of this genus is fairly similar between the two conditions. Lastly, the genera unkn. Bacteria(d), Corynebacterium, unkn. Bacillales(o), Kocuria, and unkn. Actinobacteria(c), which are among the most abundant in the control group, are not among the top genera in the KC group. This again suggests potential differences in the microbial communities between the control and KC groups.

### 3.3. Alpha Diversity Analyses Reveal Distinct Microbial Communities in Control and Keratoconus Groups

**Figure 4:**
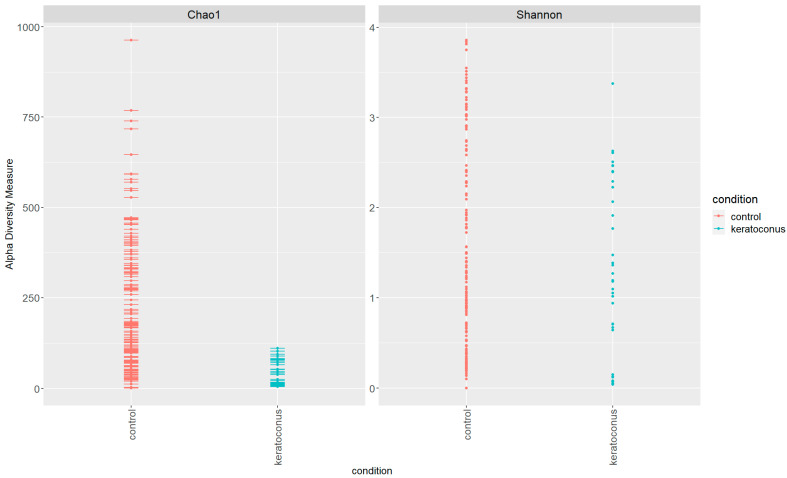
Alpha Diversity Analysis for microbial communities in the healthy control (red) and keratoconus groups (green). The analysis discloses lower microbial diversity in the KC compared with the healthy control group.

Our comprehensive analysis of the microbial communities in the healthy control and KC groups, assessed using the Chao1 richness and Shannon diversity indices (Figure 4), has uncovered significant differences. The Chao1 index, a measure of species richness, revealed that the KC group has a lower microbial richness, with most values falling between 0 and 100. Conversely, the control group demonstrated a broader distribution of Chao1 values, indicating a higher microbial richness and suggesting a more diverse and potentially more resilient microbiota. Similarly, the Shannon diversity index, which takes into account both species richness and evenness of distribution, revealed a moderate level of diversity in the KC group, while the control group displayed a higher level of diversity.

### 3.4. Principal Coordinates Analysis Reveals Distinct Microbial Community Structures in Control and Keratoconus Groups

**Figure 5:**
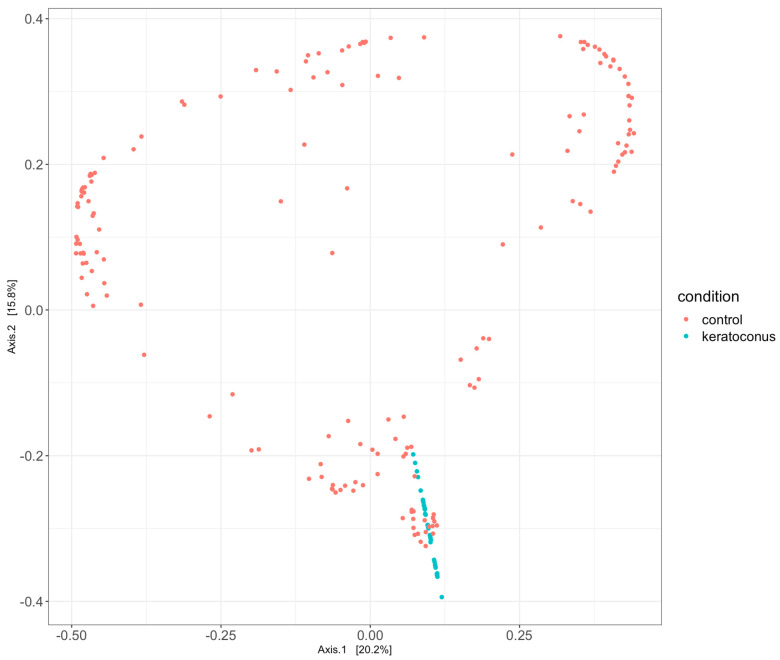
Principal Coordinates Analysis in the microbial communities, healthy control (red), and keratoconus groups (green). The investigation shows a definite representation of microbial communities between the healthy control and KC groups, suggesting a difference in sequence composition.

The Principal Coordinates Analyses (PCoA), which define a certain fraction of the variability observed in the data set, are plotted to create a visual representation of the microbial community compositional differences among samples (Figure 5).

Observations based on PCoA plots highlight differences in the microbial community structures between the control and KC groups. The PCoA plot, which visualizes the multidimensional differences in microbial community composition between samples, showed distinct clustering patterns for the two groups, suggesting a meaningful difference in the healthy control and KC microbial community.

### 3.5. Heatmap Visualization Highlights Distinct Taxonomic Markers in Control and Keratoconus Groups

**Figure 6:**
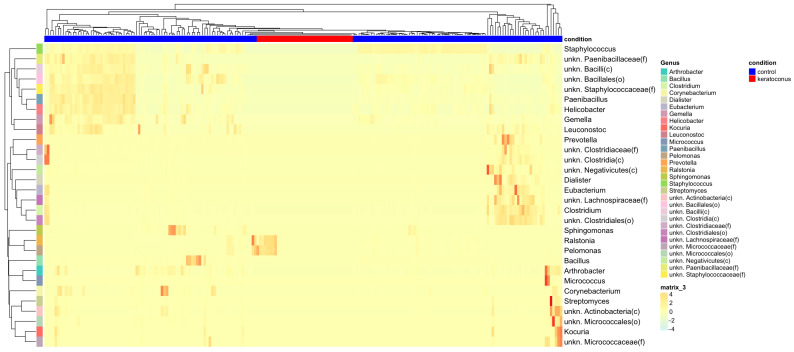
Heatmap Analysis for the taxonomic abundances in the healthy control (pink) and keratoconus groups (purple). The data display a prevalence of Ralstonia and Pelomonas taxa in the KC samples compared with healthy control samples.

A warm-to-cool color scheme was represented to visualize the taxonomic behavior data in the form of hot and cold spots (Figure 6).

The heatmap visualization of taxonomic abundance analysis shows distinct patterns of presence and absence of taxa in the control and keratoconus groups. This heatmap, which provides a color-coded representation of the relative abundances of taxa across samples, has allowed us to identify a potential taxonomic marker (Figure 6). Specifically, in the KC group, the genera Ralstonia and Pelomonas were prominently highlighted, suggesting they are prevalent in the keratoconus samples. Conversely, these genera were not observed in the control group, indicating a significant difference in the microbiota composition between the two conditions. On the other hand, the control group displayed a unique set of highlighted taxa, which were not observed in the keratoconus group. These data underscore a clear difference in the microbiota compositions of the control and KC groups, indicating a taxonomic marker.

## 4. Discussion

The pathophysiology of KC is a subject still under debate [38], with the inflammatory theory being the one that has been on the rise in recent times [39], especially due to its association with ocular rubbing [40]. However, much remains to be known since genetic factors, family associations, and systemic conditions have also been related to its development [38,41]. Moreover, the proteomic and transcriptomic analysis suggested dysregulations in oxidative stress, NRF2-regulated antioxidant programs, WNT-signaling, TGF-β, ECM, and matrix metalloproteinases as possible causes of KC [38]. Nowadays, the study of the ocular microbiome is increasing, and more and more importance is given to the possible role of the normal microbiota of the ocular flora and even the link between the gastrointestinal microbiome [42,43] in the maintenance of homeostasis as well as its imbalance in the development of different ocular pathologies [44]. Therefore, the study of the ocular microbiota in patients suffering from KC can provide a new approach when it comes to having a better knowledge of this pathology. Following this purpose, Tunç et al. [37] recently evaluated ten patients with KC who would have undergone CXL. However, it is well known that CXL is a surgical procedure that generates inflammation and remodeling of the cornea, which could theoretically influence the microbiota of these patients [45].

Our study presents a comprehensive comparison of the microbiota composition of naïve KC and a control group derived from the findings obtained in our previous research33 at various taxonomic levels, revealing distinct differences between both groups, which suggest a potential role for the microbiota in the pathogenesis of KC.

We found significant differences in the relative abundance of several phyla and genera, where the KC group showed a prevalence of the genera Ralstonia and Pelomonas, which were notably absent in the control group. Conversely, genera abundant in the control group were not found in the KC group. This distinct pattern, visible in our heatmap, suggests specific sets of taxa associated with each condition, which could potentially serve as taxonomic markers.

Pelomonas and Ralstonia are two different genera of bacteria that are known for several distinct characteristics:

- Pelomonas:
  - Habitat: Pelomonas bacteria are commonly found in aquatic environments, including freshwater, wastewater, and other water sources
  - Metabolism: These bacteria are typically aerobic and are capable of reducing nitrate to nitrite. They are also known for their ability to metabolize various organic compounds.
  - Non-Pathogenic: Pelomonas species are generally considered non-pathogenic to humans, though they have occasionally been isolated from clinical samples.
  - Role in Environmental Processes: Pelomonas bacteria play roles in various environmental processes, such as the nitrogen cycle, due to their ability to reduce nitrates.
- Ralstonia
  - Habitat: Ralstonia species are widely distributed in the environment and can be found in soil, water, and plants.
  - Pathogenicity: Some species of Ralstonia, such as Ralstonia pickettii, can be opportunistic pathogens in humans, causing infections primarily in immunocompromised individuals.
  - Plant Pathogen: Ralstonia solanacearum is a well-known plant pathogen causing bacterial wilt in a variety of plants, which can lead to significant agricultural losses.
  - Metabolic Diversity: Ralstonia species exhibit metabolic diversity and are capable of degrading a wide range of organic compounds, making them important for bioremediation.
  - Resistance: Ralstonia species are known for their resistance to heavy metals and antibiotics, which can make infections caused by these bacteria challenging to treat.
  - Biofilm Formation: Ralstonia species can form biofilms, which are communities of bacteria encased in a matrix on various surfaces. This ability can contribute to their persistence in hospital environments and medical devices.

While Pelomonas and Ralstonia share some similarities, such as being found in aquatic environments and having diverse metabolic capabilities, they differ significantly in their pathogenicity and roles in environmental and human health, and further investigations are needed to understand their role in KC.

Our alpha diversity analysis using the Chao1 richness and Shannon diversity indices demonstrated lower microbial richness and moderate diversity in the KC group, suggesting less robust microbial communities. In contrast, the control group exhibited higher microbial richness and diversity, indicative of a more resilient microbiota. This difference suggests that the KC microbiota might be less capable of withstanding disturbances, potentially making it more susceptible to imbalances that could contribute to disease progression. However, since this is a cross-sectional study, it is difficult to corroborate. A long-term longitudinal study would be needed to confirm this fact.

Our Principal Coordinates Analysis (PCoA) further underscored these significant differences, revealing distinct clustering patterns for the control and KC groups. The KC group clustered tightly, indicating a degree of similarity across samples, while the control group showed a broader distribution, reflecting more variability in microbial community structures. These findings suggest that alterations in the microbiota may play a role in the complex scenario of KC development. However, the biological implications and potential mechanisms by which these microbial communities may contribute to KC pathogenesis warrant further investigation. Longitudinal studies could provide more insight into the dynamic changes in the microbiota over the course of disease progression. We can speculate the role of microbiome in 3 points:

(1) Diagnostic: The specific composition of the microbiome can be helpful in the diagnosis of infectious diseases and monitoring microbial components of noncommunicable chronic or new-onset diseases;
(2) Risk Factor: The microbiome can be involved in the understanding of disease progression. Some ECST KC microbiomes could be linked to quicker KC progression;
(3) Therapeutic: understanding the role of microbiome in KC patients could change the choices of current treatments and/or help develop new treatments focused on KC microbiome improvements.

Furthermore, microbiome functional analyses, such as metatranscriptomic or metabolomic studies, could shed light on the functional implications of these alterations in the microbiome and microbiota.

Ultimately, a better understanding of the role of the microbiome and microbiota in KC could lead to novel diagnostic or therapeutic strategies. Specifically, eye microbiome screening tests could become routine evaluations to check for potential diseases before signs appear. Developing ECST for specific diseases could ease the comparison process with healthy subjects. Also, the management of KC patients could be influenced by demonstrating that specific KC ECTS could be linked to a faster disease progression and other to no or slow progression. Additionally, the differences in microbiota in the KC and control groups do not prove causation. Further studies need to evaluate where microbiome changes could cause KC disease or where KC could influence the microbiome.

Low microbiota diversity can potentially impact KC patients in various ways, as the microenvironment of the eye plays a critical role in maintaining ocular health. Here are some possible micro-environmental impacts:

- Increased Susceptibility to Infections: Lower diversity in microbiota might make the ocular surface more susceptible to pathogenic infections due to reduced competition for resources and colonization sites.
- Altered Immune Response: The diversity of microbiota contributes to the modulation of local immune responses. A decrease in diversity could alter immune regulation, potentially leading to increased inflammation or autoimmune reactions, which could exacerbate KC progression.
- Changes in Metabolite Production: Microbiota produce various metabolites that can influence host tissue. A reduction in microbiota diversity could alter the profile of these metabolites, affecting the nutritional and metabolic environment of the cornea, which might impact KC.
- Disruption of Homeostasis: Microbial diversity is essential for maintaining homeostasis on body surfaces, including the eye. Reduced diversity might disrupt the balance between the host and microbiota, potentially leading to ocular surface disease and affecting the progression of KC.
- Impact on Treatment Response: A less diverse microbiome may impact how patients respond to treatments, as the microbiota can affect drug metabolism and modulate host responses.
- Development of Antibiotic Resistance: Low diversity can result in domination by a few species, leading to antibiotic resistance development and making infections harder to treat.
- Modification of Tear Film Composition: The microbiota can influence the composition of the tear film. Changes in microbiota diversity might affect tear film stability and composition, contributing to ocular surface abnormalities.

Research is ongoing in this area, and further studies are necessary to fully understand the specific micro-environmental impacts of low microbiota diversity in KC patients and how these changes might contribute to disease progression or offer therapeutic targets.

The study has a few limitations. First, the study design is cross-sectional, meaning it only provides a snapshot in time of the microbiota compositions in the KC and control groups. This design limits the ability to make causal inferences regarding the role of microbiota in the progression or onset of KC. Longitudinal studies would be better suited to trace the dynamics of microbiota changes over time and their potential impact on disease progression. Second, the sample size difference between the control and KC groups could influence Alpha diversity and increase as the sample size increases.

Additionally, the study does not mention any adjustments for potential confounding factors, such as age, gender, diet, lifestyle, environmental factors, or the potential changes of microbiome after contact lens suspension. Any of these factors could influence the composition of the microbiota, thereby impacting the observed differences between the KC and control groups.

Limited to Taxonomic Analysis, while the study provides detailed taxonomic analysis, it does not delve into the functional roles of the identified microbial communities. Functional analysis, such as metatranscriptomic or metabolomic studies, would provide a more comprehensive understanding of how the microbiota might influence or be influenced by KC.

## Data Availability

Data sharing does not apply to this article. No new data were created or analyzed in this study.
